# Strategies to Target Microbial Attack in Chronic Skin Wounds: From Classic to Innovative Approaches

**DOI:** 10.3390/bioengineering10060666

**Published:** 2023-06-01

**Authors:** Cláudia S. Oliveira, Rossella Laurano

**Affiliations:** 1 CBQF-Centro de Biotecnologia e Química Fina–Laboratório Associado, Escola Superior de Biotecnologia, Universidade Católica Portuguesa, Rua Diogo Botelho 1327, 4169-005 Porto, Portugal; 2 Department of Mechanical and Aerospace Engineering, Politecnico di Torino, Corso Duca degli Abruzzi 24, 10129 Turin, Italy; rossella.laurano@polito.it

Chronic skin wounds, namely diabetic/non-diabetic ulcers and post-surgical wounds, present key obstacles to achieve anatomic and functional regeneration within approximately 3 months [1]. Such skin injuries fail to heal in an orderly and timely reparative process because they do not overcome the inflammatory phase or impair the proliferation of endothelial cells and fibroblasts, as well as collagen matrix deposition. In addition, chronic wounds are also characterized by an imbalance between pro- and anti-inflammatory cytokines and growth factors at the wound site, which hinder the wound healing mechanism [2]. Irrespective of their origin, chronic wounds have the following common features: (i) excessive levels of pro-inflammatory cytokines; (ii) high levels of proteases that cause the destruction of extracellular matrix (ECM) and the degradation of growth factors; (iii) excessive release of reactive oxygen species (ROS) via inflammatory cells which induce oxidative damage in cells and the ECM; (iv) a deficiency of stem cells for an appropriate skin regeneration; and (v) a high risk of persistent infections [3].

Not surprisingly, such a prolonged inflammatory environment is an easy target for microbial attack, which usually results in biofilm formation via microbial aggregation. As a consequence, the presence of biofilm significantly delays the healing process due to its high resistance to conventional therapies [4]. Therefore, microbial attack and biofilm formation have a negative impact on the pathogenesis of chronic wounds, leading to impaired tissue healing and significantly compromising the quality of life for many patients.

In wound management, the control of infections represents a crucial issue and is a multi-billion-dollar industry worldwide. For instance, infections are responsible for the majority of amputations in diabetic patients with chronic ulcers, which currently represents an increasing number of the elderly [5]. Unfortunately, the gold-standard treatment for wound infections, i.e., the administration of high dosages of antibiotics, topical or oral, still presents disadvantages, including (i) undesired side effects on non-target tissue; (ii) a negative impact on the resident microbiota members of skin; (iii) the increase in antibiotic resistance, (iv) the necessity for prolonged treatments; and (iv) the risk of infection dissemination. Therefore, to overcome these critical concerns, the design of innovative, efficient, and antibiotic-free treatments has become increasingly important to promptly tackle the negative impact that microbial attacks and infections exert on wound healing (Figure 1). In this scenario, naturally derived compounds, and properly engineered biomaterials [6,7,8] have received special attention as antimicrobial alternatives due to their beneficial biological outcomes (i.e., anti-inflammatory properties and their capability to reduce oxidation and microbial invasions).

In this sense, the purpose of this Special Issue is to provide the reader with an overview of the most imperative strategies able to target microbial attack in chronic skin wounds. For this, contributions from classic to advanced and innovative approaches will be explored. Additionally, particular interest will be devoted to works dealing with the engineering of new drug delivery carriers (e.g., smart/biofunctional hydrogels, nanosystems, membranes, textile materials, and dressings) with specific antimicrobial/anti-inflammatory properties. Personalized therapies approaches involving advanced fabrication technologies and green chemistries to give value to waste- and agricultural-derived biomaterials (e.g., alginate, chitosan, zein, and hyaluronic acid) will also receive special attention. Furthermore, papers exploring natural compounds able to act as therapeutic agents (e.g., polyphenols) are highly recommended. Therefore, this Special Issue expects to provide not only a discussion about new alternatives/strategies for the treatment, control, and management of infected wounds but also open horizons for the use and development of more sustainable therapeutic approaches through the stimulation of circular (bio) economy models.

## Figures and Tables

**Figure 1:**
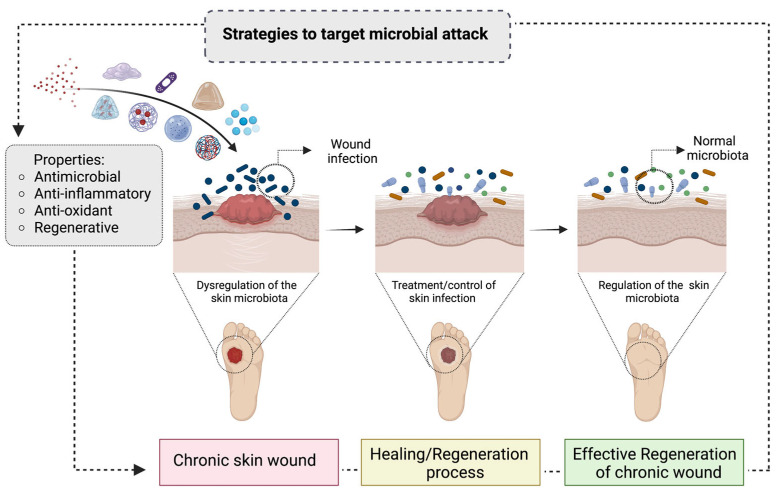
Schematic representation of the potential of strategies to target microbial attack in chronic skin wounds. The image highlights the design of different strategies able to deliver key biological properties, namely antimicrobial, anti-inflammatory, antioxidant, and regenerative, to chronic skin wounds. Locally, these strategies should first fight wound infections and modulate the skin microbiota. These events could decrease the negative impact of microbial attacks and infections while facilitating the healing and regeneration process of chronic skin wounds.
